# Supplementary material for: Silica-Polymer Composites as the Novel Antibiotic Delivery Systems for Bone Tissue Infection
Source: Pharmaceutics. 2019 Dec 30;12(1):28. doi: 10.3390/pharmaceutics12010028 (PMC7022428; doi:10.3390/pharmaceutics12010028)
Supplement: Supplementary file 1 [file pharmaceutics-12-00028-s001.pdf]

pharmaceutics

Supplementary Materials: Silica-Polymer Composites as the Novel Antibiotic Delivery Systems for Bone Tissue Infection

Adrianna Skwira, Adrian Szewczyk, Agnieszka Konopacka, Monika Górska, Dorota Majda, Rafał Sądej and Magdalena Prokopowicz

Table S1. Surface area, pore diameter and pore volume of MCM-41 and MCM-41-CIP powders.

Sample	Surface Area (S_{BET}) (m^2/g)	Pore Diameter (nm)	Pore Volume (cm^3/g)
MCM-41	720	3.55	0.61
MCM-41-CIP	590	2.98	0.47

Note: calculated from the desorption branch of the nitrogen adsorption-desorption isotherm using the Barrett–Joyner–Halenda (BJH) method.

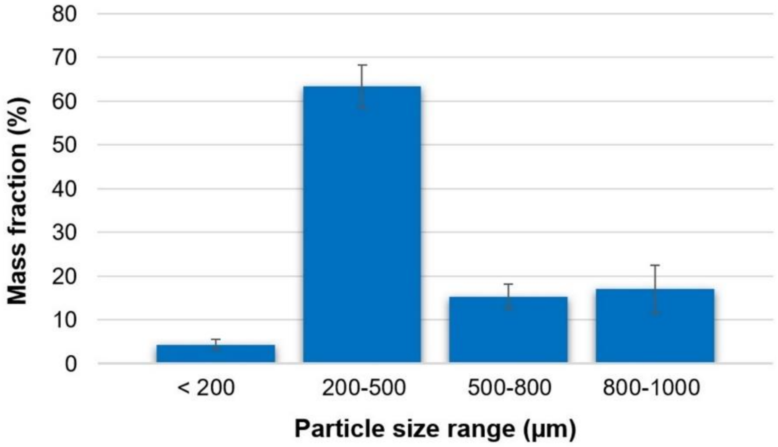

Figure S1. Particle size of MCM-41-CIP powder.

Table S2. The kinetic parameters of fitted experimental data for MCM-41-CIP sample and EC/CIP, EC/PDMS/CIP, EC/MCM-41-CIP and EC/PDMS/MCM-41-CIP composites.

Sample	Higuchi Model		Korsmeyer-Peppas Model	
	k_H	R^2	n	R^2
MCM-41-CIP [1]	7.44	0.882	0.42	0.911
EC/CIP [1]	27.6	0.952	0.50	0.954
EC/PDMS/CIP [1]	30.4	0.936	0.54	0.951
EC/MCM-41-CIP [2]	7.39	0.992	0.17	0.979
EC/PDMS/MCM-41-CIP [3]	2.68	0.993	0.26	0.866

R^2—coefficient of determination, n—release exponent in Korsmeyer-Peppas model, k_H—Higuchi dissolution constant ($min^{-1/2}$; $day^{-1/2}$). [1] calculated for 60% of cumulative amount of released CIP; [2] calculated for first 11 days of release study (60% of cumulative amount of released CIP); [3] calculated for 30 days of release study (21% of cumulative amount of released CIP).
